# Effects of Starvation and Refeeding on Growth, Digestion, Nonspecific Immunity and Lipid-Metabolism-Related Genes in *Onychostoma macrolepis*

**DOI:** 10.3390/ani13071168

**Published:** 2023-03-25

**Authors:** Nina Gou, Kaifeng Wang, Tiezhi Jin, Bin Yang

**Affiliations:** Shaanxi Institute of Zoology, Xi’an 710032, China

**Keywords:** lipid metabolism, nonspecific immunity, *Onychostoma macrolepis*, refeeding, starvation

## Abstract

**Simple Summary:**

Starvation is likely to occur in all animals. Many fish species have demonstrated the ability to withstand short- or long-term starvation, and *Onychostoma macrolepis* is one of them. *O. macrolepis* is a national second-class protected animal in China, which has the dual value of protection and utilization. The main objective of this research was to characterize the physiological and metabolic changes in *O. macrolepis* during starvation and refeeding. The results indicated that fish growth was inhibited during fasting and improved after refeeding. *O. macrolepis* could respond to changes in nutritional status by changing their antioxidant capacity. When food was scarce, the consumption of fat in fish contributed to sustain the fundamental energy requirements. Conversely, the reintroduction of the food supply could restore lipid levels. These findings could provide a reference for understanding the adaptive strategies of fish, particularly *O. macrolepis*, during starvation and refeeding.

**Abstract:**

The present research was conducted to assess the influences of starvation and refeeding on growth, nonspecific immunity and lipid metabolic adaptation in *Onychostoma macrolepis.* To date, there have been no similar reports in *O. macrolepis*. The fish were randomly assigned into two groups: control group (continuous feeding for six weeks) and starved–refed group (starvation for three weeks and then refeeding for three weeks). After three weeks of starvation, the results showed that the body weight (BW, 1.44 g), condition factor (CF, 1.17%), visceral index (VSI, 3.96%), hepatopancreas index (HSI, 0.93%) and intraperitoneal fat index (IPFI, 0.70%) of fish were significantly lower compared to the control group (BW, 5.72 g; CF, 1.85%; VSI, 6.35%; HSI, 2.04%; IPFI, 1.92%) (*p* < 0.05). After starvation, the serum triglyceride (TG, 0.83 mmol/L), total cholesterol (T-GHOL, 1.15 mmol/L), high-density lipoprotein (HDL, 1.13 mmol/L) and low-density lipoprotein (LDL, 0.46 mmol/L) concentrations were significantly lower than those in the control group (TG, 1.69 mmol/L; T-GHOL, 1.86 mmol/L; HDL, 1.62 mmol/L; LDL, 0.63 mmol/L) (*p* < 0.05). The activities of intestinal digestive enzymes (amylase, lipase and protease) in the starved-refed group were significantly lower than those in the control group after three weeks of starvation (*p* < 0.05). The highest activities of immune enzymes such as lysozyme (LZM), acid phosphate (ACP), alkaline phosphate (ALP), superoxide dismutase (SOD), glutathione peroxidase (GSH-PX) and catalase (CAT) in the hepatopancreas were presented in the starved–refed group at second week, and significantly higher than those in the control group (*p* < 0.05). Meanwhile, starvation significantly improved intestinal immune enzymes activities (*p* < 0.05). the lowest TG contents and the highest expression levels of lipolysis genes including hormone-sensitive lipase (HSL) and carnitine palmitoyl transferase 1 isoform A (CPT-1A) appeared in the hepatopancreas, muscle and intraperitoneal fat after starvation, indicating the mobilization of fat reserves in these tissues (*p* < 0.05). After refeeding, the recovery of TG content might be mediated by the upregulation of the expression levels of lipogenesis genes such as sterol regulatory element binding protein 1 (SREBP1) and fatty acid synthase (FAS). Understanding the duration of physiological and metabolic changes in *O. macrolepis* and their reversibility or irreversibility to supplementary feeding response could provide valuable reference for the adaptability of *O. macrolepis* in large-scale culturing, proliferation and release.

## 1. Introduction

Food restriction is likely to occur in all animals due to numerous factors such as population competition, unequal spatial distribution of food, seasonal variations and ecological instability [1,2]. Unlike other vertebrates, many fish species have demonstrated the ability to withstand short- or long-term starvation [3]. During starvation, changes in the depletion of endogenous energy reserves, immune status and the expression of genes involved in different metabolic pathways are all induced in fish [4,5,6]. To maintain important aspects of survival and movement during starvation, fish have to reallocate their energy storage, especially lipids [7,8]. Lipids are the main energy source for fish to maintain their fundamental metabolism during starvation, and proteins are mobilized only when necessary [9]. During a period of food scarcity, when stored lipid energy in fish is used as a fuel resource, multiple intermediate pathways of nutrient metabolism can be induced. Several lipid metabolites including fatty acids, cholesterol and triglyceride (TG) have been evaluated to speculate about the physiological adaptation of fish [10]. Nevertheless, the levels of various lipid metabolites were not consistent between different species during starvation.

Starvation for 4 days and feeding for 16 days (three cycles for a total of 60 days) increased the plasma TG concentration in rainbow trout (*Oncorhynchus mykiss*) (17.5 ± 0.06 g) [11]. However, eight weeks of starvation decreased the TG levels in the tissues of grass carp (*Ctenharyngodon Idellus*) (2252.00 ± 37.88 g) [12]. Two weeks of fasting induced a significant reduction in cholesterol content in tinfoil barb (*Barbonymus schwanenfeldii*) (16.20 ± 0.13 g), while there was no significant difference found in the cholesterol level of Gibel carp (*Carassius auratus gibelio*) (111.13 ± 0.65 g) after 21 days of fasting [13,14]. After four weeks of starvation, the plasma cholesterol and TG of the great sturgeon (*Huso huso*) (78.27 ± 0.2 g) decreased significantly [15]. These studies suggested that starvation had different effects on TG and cholesterol levels in fish due to interactions of species differences, environmental factors and fasting duration. For these reasons, the potential relationship between food deprivation and changes in the lipid metabolites levels is unclear. Accordingly, there have been few studies conducted to evaluate how starvation regulates the expression levels of genes related to lipid metabolism in fish [14,16]. *Onychostoma macrolepis* is a national second-class protected animal in China, which has the dual value of protection and utilization. As a high-value food, it is very popular for its delicious taste and rich nutrition. This fish is resistant to stress and can be cultured at a high stocking rate. *O. macrolepis* culturing has begun to take shape and has developed steadily in China. At present, there has been no report on the regulation of lipid metabolism genes expression during short-term starvation and refeeding in *O. macrolepis*. 

Apart from the energy mobilization process, starvation also affects the digestion and nutrient absorption of fish. Previous research studies showed that fish could cope with starvation stress by regulating the digestive enzymes activity [17]. In Nile tilapia (*Oreochromis niloticus*) (45.08 ± 0.92 g), the digestive enzymes activities reduced significantly after three weeks of fasting and rose again when the feeding was resumed for three weeks [18]. Several studies involving different feeding regimes reported that starvation duration could appreciably impact activities of digestive enzymes in hybrid grouper (*Epinephelus fuscoguttatus*♀ × *E. lanceolatus*♂) and Songpu mirror carp (*Cyprinus carpio* L.) [19,20]. Therefore, it is of great significance to further study the variations of intestinal digestive enzyme activities during fasting and refeeding. Moreover, starvation influences the immune reaction of fish by changing biological processes and metabolic characteristics [21]. A recent review by American scholars suggested that fasting could improve oxidative stress in fish [22]. Malondialdehyde (MDA), a biomarker of oxidative stress, is one of the final products of lipid peroxidation. The excessive accumulation of reactive oxygen species (ROS) has adverse impacts on cellular structures and components. Enzymatic and nonenzymatic antioxidant eliminating systems could sustain a lower level of ROS in fish. It is well-known that superoxide dismutase (SOD), glutathione peroxidase (GSH-PX) and catalase (CAT) are several important antioxidant enzymes. Antioxidant enzymes play a vital role in scavenging free radicals and regulating the generation of ROS, thereby activating the antioxidant defense mechanism and further improving the immune status in fish [23]. Previous studies reported that 14 days of starvation could elevate antioxidant enzymes activities in fish such as the Chinese perch (*Siniperca chuatsi*) (250 ± 22 g) and Stellate Sturgeon (*Acipenser stellatus*) (331.3 ± 71.8 g) [24,25]. In *Siniperca chuatsi* (562.20 ± 31.41 g), the activity of antioxidant enzyme remarkably strengthened after three days of starvation [26]. On the contrary, another research showed that the activities of antioxidant enzymes were significantly decreased in the liver of the Ganus golden trout (*Oncorhynchus mykiss*) (54 ± 0.51 g) after 21 or 28 days of starvation [27]. These findings indicated that starvation had dramatic effects on immune regulation in fish. 

From the perspective of researchers and fish farmers, short-term starvation was usually feasible if it had no adverse effect on fish growth [28]. This feeding method was beneficial to conserve feed and labor costs to improve farm profits. Previous studies used various fasting and refeeding methods to detect the growth performance and metabolic characteristics of different fish species such as the brown trout (*Salmo trutta*) and the Nile tilapia [16,18]. These studies found that when food supplies were restored, the hungry fish experienced unusually rapid growth rates, called compensatory growth [7]. In addition to the powerful evidence of a recovery in growth performance, compensatory growth is a response to the restoration of lipid levels [7]. Several researchers attempted to exploit the compensatory growth of aquaculture fish species such as the Atlantic salmon to develop appropriate feeding regimes, with success [29]. This study investigated the influences of starvation and refeeding on the growth, digestion, nonspecific immunity and lipid metabolism in *O. macrolepis*. To date, there have been no similar reports in *O. macrolepis*. Thus, the research on the adaptive response of *O. macrolepis* during starvation and refeeding is novel. It is helpful to better understand the adaptability of the fish to large-scale culture, proliferation and release.

## 2. Materials and Methods

### 2.1. Ethics Statement

This experiment followed the Guidelines for the Care and Use of Laboratory Animals in China and animal welfare and ethical codes. All efforts were made to minimize animal suffering. The study was conducted according to the guidelines of the Declaration of Helsinki and approved by the Animal Ethics Committee of Shaanxi Institute of Zoology (Approval No. L22D005A51).

### 2.2. Fish Maintenance and Experimental Conditions

The starvation and refeeding experiment was carried out at Shaanxi Institute of Zoology (Xi’an, China) and lasted for six weeks. *O. macrolepis* fish were purchased from Laowangfu fish farm (Tai’an, China). 

These fish were acclimated for two weeks at room temperature with a natural photoperiod to adapt to the experimental environment. During the trial, the experimental fish were fed a commercial formula diet until satiation (Table 1).

According to different feeding methods, 120 fish (initial weight: 2.50 ± 0.14 g and 2.51 ± 0.17 g) were randomly selected and assigned into two groups with six replicates, named as the control group (continuous feeding for six weeks) and starved–refed group (starvation for three weeks and then refeeding for three weeks). The experiment consisted of 12 aquariums (50 cm, 30 cm and 45 cm; length, width and height) each containing ten fish. In the control group, the fish were kept fed until satiety three times a day at 8:30, 12:30 and 16:30, with a commercial diet throughout the experimental time. The fish in the starved–refed group maintained the same feeding pattern as the control fish during the refeeding period after starvation.

The fish waste and remaining feed were scavenged daily using siphoning. One third of the water was renewed every three days, and the quality parameters were as follows: temperature, 20 ± 1 °C; pH value, 7.0 ± 0.5; dissolved oxygen, 6.5 ± 0.5 mg/L (mean ± SD).

### 2.3. Sample Collection

The samples were collected once a week for six times in total during the experiment, with weeks 1, 2 and 3 corresponding to the starvation period and weeks 4, 5 and 6 to the refeeding period. The fish (one fish per aquarium, six fish per treatment) were randomly chosen and euthanized with MS-222 (60 mg/L) before sampling. The body weight of each individual was measured with an electronic balance. A blood sample was drawn from the caudal vessels using a heparin syringe, left at 4 °C for 6 h and centrifuged to collected supernatant (3000 r/min, 4 °C, 10 min). The serum was immediately frozen with liquid nitrogen and stored at −80 °C for subsequent biochemical testing. The fish were dissected and the viscera, hepatopancreas and intraperitoneal fat samples were removed and weighed separately. The same six fish were used for body weight recording, blood collection and dissection. The samples of hepatopancreas, intestines, muscle and intraperitoneal fat were rapidly isolated and stored at −80 °C for biochemical and molecular detection (six fish per treatment).

### 2.4. Serum Parameters’ Analysis

The contents of TG, total cholesterol (T-CHOL), high-density lipoprotein (HDL) and low-density lipoprotein (LDL) in serum were analyzed by a TBA-120FR automatic biochemical analyzer (Toshiba Co., Ltd., Tokyo, Japan). 

### 2.5. Determination of Digestive Enzymes, Immune Activities and TG Contents in Tissues

The samples of tissue were homogenized with a tissue homogenizer in nine volumes of ice-cold saline solution and the entire procedure was performed on ice.

The mixtures were centrifuged for 10 min (4 °C, 3000 r) and the supernatants were obtained for the subsequent testing. Then, 1 mL supernatant was taken and transferred into a centrifuge tube for subsequent detection. The tissues’ protein content was analyzed using a BCA total protein assay kit (A045-4) (Nanjing Jiancheng, Nanjing, China). The amylase (C016-1-1), lipase (A054-1) and protease (A080-2) activities in intestines were measured by the assay kits of the Nanjing Jiancheng Bioengineering Institute according to the manufacturer‘s instructions. The lysozyme (LZM) (A050-1-1), acid phosphate (ACP) (A060-1-1), alkaline phosphate (ALP) (A059-1-1), SOD (A001-1), GSH-PX (A005-1), CAT (A007-2-1) activities and MDA (A003-1) content in the hepatopancreas and intestines were determined by special kits (Nanjing Jiancheng Bioengineering Institute, Nanjing, China). The TG assay kits (A110-2-1) were purchased from the same company and were used to measure TG contents in hepatopancreas, muscle and intraperitoneal fat. All detection process were conducted by strictly following the instructions of the kits. Firstly, distilled water was added into the blank tube, the standard substance was added into the standard tube, and the sample was added into the sample tube and then incubated according to the temperature and time required by the kit. Next, the wavelength of the spectrophotometer was set, and the OD value was read. Finally, the results were calculated according to the formula in the instructions of the assay kits.

### 2.6. Gene Expression Analysis

The expression of related genes was detected by real-time quantitative PCR using the method reported by Gou et al. [30]. The qPCR primers for hormone-sensitive lipase (HSL), carnitine palmitoyl transferase 1 isoform A (CPT-1A), sterol regulatory element binding protein 1 (SREBP1), fatty acid synthase (FAS) and β-actin were designed using Primer Express 5.0, as shown in Table 2. Three technical replicates were detected for each sample according to the kit’s instructions. Briefly, the amplification reaction solution consisted of 10 μL 2 × ChamQ SYBR qPCR Master Mix (Vazyme), 0.4 μL 50 × ROX reference dye 2, 0.4 μL of each primer (10 μM/L), 1 μL cDNA and 7.8 μL aseptic double-distilled water in a final volume of 20 μL. 

The ABI 7500 Real-time PCR system was employed for the amplification (Applied Biosystems Inc., Waltham, USA). The reaction conditions were: 95 °C for 30 s, 40 cycles of 95 °C for 10 s and 60 °C for 30 s. The target gene expression levels were computed by following the 2^−ΔΔCt^ method [31].

### 2.7. Statistical Analysis

Data processing was performed using SPSS 22.0 software. All data are presented as mean ± standard deviation of at least three independent experiments. A two-way analysis of variance (ANOVA) was used to assess significant differences in feeding treatment factors (nutritional condition and time). *p* < 0.05 was considered for the confidence level.

## 3. Results

### 3.1. Growth Performance

The growth indicators of fish in the control and the starved–refed groups were significantly affected by the food supply (*p* < 0.05) (Table 3). No mortality occurred in the two experimental groups during the trial. At the end of the six-week trial, the body weight (BW), condition factor (CF), visceral index (VSI), hepatopancreas index (HSI) and intraperitoneal fat index (IPFI) of fish in the starved–refed group were significantly lower than those in the control group (*p* < 0.05). There were significant differences in growth and biological indicators between the control group and the starved-refed group during starvation (*p* < 0.05). With the starvation time prolonged, the BW, CF, VSI, HSI and IPFI of fish were significantly reduced in the starved-refed group compared with the control group (*p* < 0.05). In the refeeding phase, there were significant differences appearing in the BW, CF, VSI, HSI and IPFI values between the control and starved–refed groups (*p* < 0.05). The various growth indicators were all shown to be positively correlated with the refeeding time. These results indicated that fish growth was inhibited during starvation and improved after refeeding.

### 3.2. Serum Parameters

Starvation and refeeding had significant impacts on serum parameters in *O. macrolepis* (*p* < 0.05) (Table 4). After three weeks of starvation, the concentrations of TG, T-CHOL, HDL and LDL were significantly lower than those in the control group (*p* < 0.05). With the refeeding time extended, the levels of TG, T-CHOL, HDL and LDL in the starved-refed group presented a distinct upward tendency (*p* < 0.05). After three weeks of re-feeding, the TG and T-CHOL levels had no significant difference between the starved-refed group and the control group. In terms of serum lipids, a three-week refeeding phase was discovered to be sufficient to protect the starved–refed fish from the adverse effects of the three-week starvation.

### 3.3. Digestive Enzyme Activities

The effects of starvation and refeeding on the intestinal digestive activities of *O. macrolepis* were significantly different (*p* < 0.05) (Table 5). The measured results of the digestive enzyme showed that the activities of amylase, lipase and protease in the starved–refed group were significantly different from those in the control group during the starvation phase (*p* < 0.05). As the duration of starvation, the activities of these three enzymes were significantly decreased in the starved–refed group compared to the control group (*p* < 0.05). After refeeding, there were no statistical differences discovered in the amylase and lipase activities between the starved–refed and control groups.

### 3.4. Immune Enzyme Activities

In comparison with the fish in the control group, the fish in the starved–refed group presented significantly higher LYZ, ACP and ALP activities in the hepatopancreas and intestines (*p* < 0.05) (Table 6 and Table 7). The hepatic LYZ, ACP and ALP activities in the starved–refed group increased first and then decreased with the duration of starvation, and the activities of these three enzymes all reached the highest values in the second week (*p* < 0.05). After three weeks of starvation, the LYZ, ACP and ALP activities in the intestines increased significantly in the starved–refed group compared to the control group (*p* < 0.05). After three weeks of refeeding, there were no significant differences discovered in the ALP activity of the hepatopancreas and intestines between the starved–refed and control groups.

### 3.5. Antioxidant Indices

The antioxidant indices in the hepatopancreas and intestines of *O. macrolepis* in the starved–refed group were significantly affected by starvation and refeeding (*p* < 0.05) (Table 8 and Table 9). With the extension of starvation time, the activities of antioxidant enzymes in the hepatopancreas of fish tended to first increase and then decrease and showed their highest levels at the second week. After three weeks of starvation, the activities of antioxidant enzymes including SOD, GSH-PX and CAT in the hepatopancreas and intestines in the starved–refed group were significantly higher than those in the control group (*p* < 0.05). Meanwhile, the MDA levels in the hepatopancreas and intestines were significantly decreased in the starved–refed group compared with the control group (*p* < 0.05). During the refeeding period, the antioxidant enzymes’ activities both in the hepatopancreas and intestines presented a downward trend.

### 3.6. Lipid Metabolic Genes Expression and TG Contents in Hepatopancreas, Muscle and Intraperitoneal Fat

**Figure 1:**
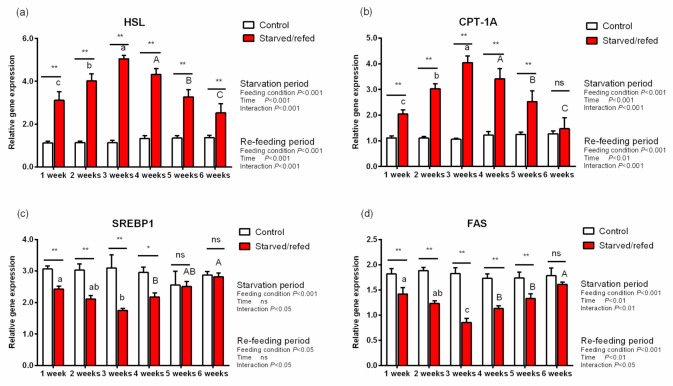
The expression of lipid metabolism genes in the hepatopancreas of *O. macrolepis* exposed to starvation and refeeding periods. Different superscript letters in the same column indicate a significant difference between successive times (*p* < 0.05). Significant differences between the two groups at each sampling time are identified with different markers (* *p* < 0.05; ** *p* < 0.01; ns, no significant difference). HSL (**a**), hormone-sensitive lipase; CPT-1A (**b**), carnitine palmitoyl transferase 1 isoform A; SREBP1 (**c**), sterol regulatory element binding protein 1; FAS (**d**), fatty acid synthase.

**Figure 2:**
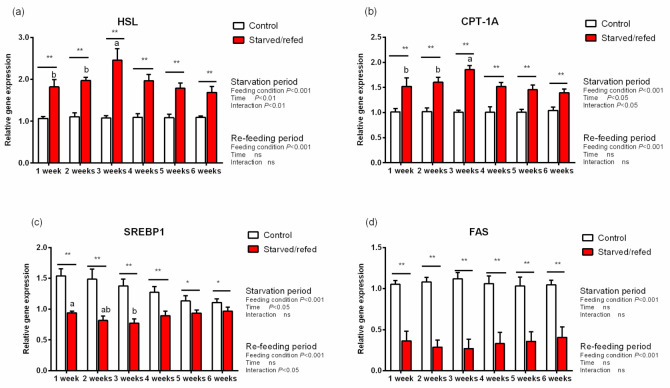
The expression of lipid metabolism genes in the muscle of *O. macrolepis* exposed to starvation and refeeding periods. Different superscript letters in the same column indicate a significant difference between successive times (*p* < 0.05). Significant differences between the two groups at each sampling time are identified with different markers (* *p* < 0.05; ** *p* < 0.01; ns, no significant difference). HSL (**a**), hormone-sensitive lipase; CPT-1A (**b**), carnitine palmitoyl transferase 1 isoform A; SREBP1 (**c**), sterol regulatory element binding protein 1; FAS (**d**), fatty acid synthase.

**Figure 3:**
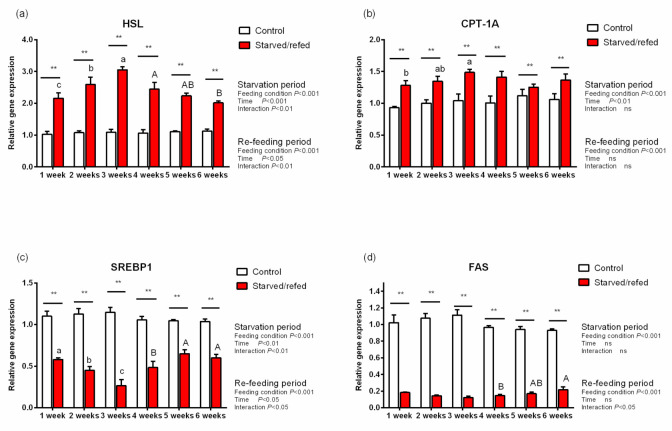
The expression of lipid metabolism genes in the intraperitoneal fat of *O. macrolepis* exposed to starvation and refeeding periods. Different superscript letters in the same column indicate a significant difference between successive times (*p* < 0.05). Significant differences between the two groups at each sampling time are identified with different markers (** *p* < 0.01; ns, no significant difference). HSL (**a**), hormone-sensitive lipase; CPT-1A (**b**), carnitine palmitoyl transferase 1 isoform A; SREBP1 (**c**), sterol regulatory element binding protein 1; FAS (**d**), fatty acid synthase.

**Figure 4:**
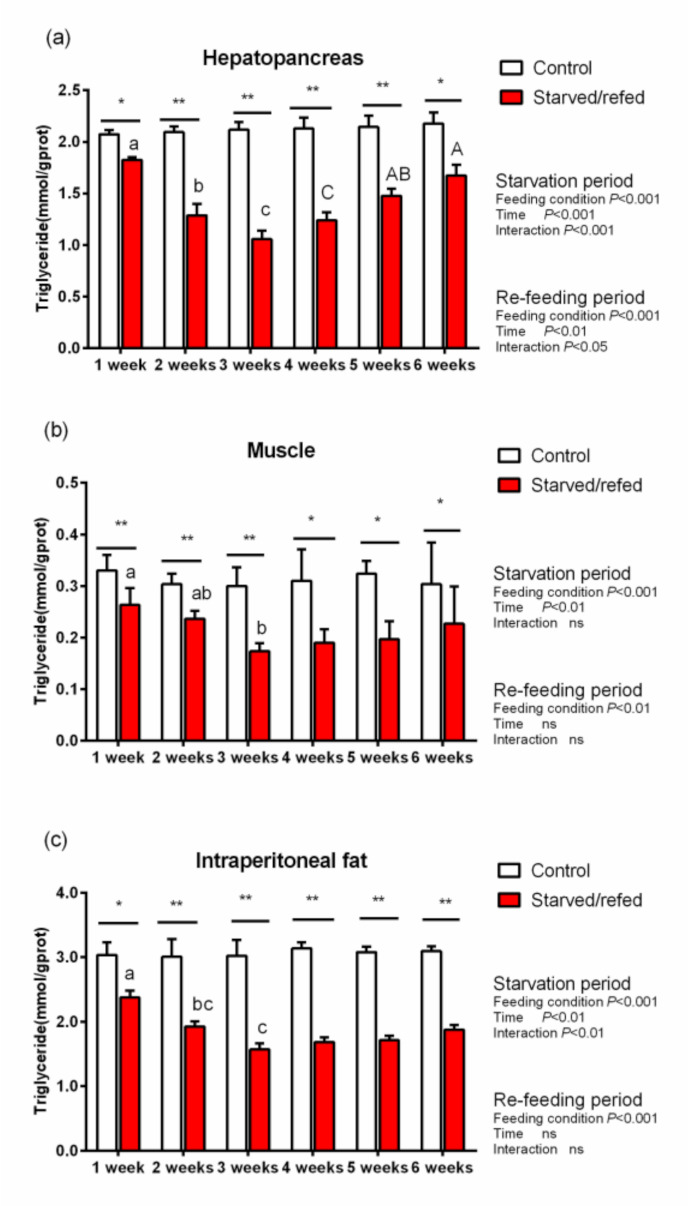
The triglyceride (TG) contents in the hepatopancreas (**a**), muscle (**b**) and intraperitoneal fat (**c**) of *O. macrolepis* exposed to starvation and refeeding periods. Different superscript letters in the same column indicate a significant difference between successive times (*p* < 0.05). Significant differences between the two groups at each sampling time are identified with different markers (* *p* < 0.05; ** *p* < 0.01; ns, no significant difference).

During starvation, the expression of lipid metabolism genes and TG contents in the hepatopancreas, muscle and intraperitoneal fat were significantly different between the control group and the starved–refed group (*p* < 0.05). With regards to the hepatopancreas muscle and intraperitoneal fat, the expression of HSL and CPT-1A in the starved–refed group increased significantly compared to the control group as the starvation time prolonged, while the expression levels of SREBP1 and FAS exhibited an opposite trend (*p* < 0.05) (Figure 1, Figure 2 and Figure 3). After refeeding, there were no significant differences found in the relative expression levels of SREBP1 and FAS in the hepatopancreas between the starved–refed and control groups. After three weeks of starvation, the lowest contents of TG in the hepatopancreas, muscle and intraperitoneal fat were observed in the starved–refed group, and they were significantly lower in this group than in the control group (*p* < 0.05) (Figure 4). After three weeks of refeeding, TG levels in tissues showed an increasing trend.

## 4. Discussion

These results provided a valuable reference for studying the reversibility or irreversibility of physiological metabolism changes in *O. macrolepis* under different nutritional conditions. Starvation, an integral part of the life history in many species, was reported to have noticeable effects on the growth, oxidative stress and metabolism of fish [22,32]. This study showed that fish growth was inhibited during starvation and then improved after refeeding. The results were in agreement with previous reports, demonstrating that four weeks of food deprivation could depress body weight in the Yangtze sturgeon (*Acipenser dabryanus*) (193.67 ± 30.75 g) [5], great sturgeon (78.27 ± 0.2 g) [15] and blunt snout bream (8.20 ± 0.01 g) [33], while four weeks of refeeding could restore the weight of Yangtze sturgeon. Weight loss in fish might be due to the reduced metabolic capacity of tissues and the decomposition of energy reserves required to maintain the endogenous homeostasis induced by starvation [34]. It was reported that the raring pattern of fasting and refeeding could induce a compensatory growth of fish, that is, a period of malnutrition followed by an accelerated growth when favorable conditions were returned [7]. In the current study, *O. macrolepis* in the starved–refed group gained weight after refeeding, indicating that a growth compensation occurred after the food reintroduction. The result was in accordance with previously reported ones, showing that a compensatory growth could occur in Nile tilapia (96.44 ± 0.49 g) after 21 days of refeeding, and in hybrid grouper (74.16 ± 12.08 g) after 30 days of refeeding, accompanied by a weight gain [19,32]. The condition factor is not only a basic index to measure the energy reserve level of fish but also an important parameter of health status [35]. In the present study, the CF value of fish decreased significantly during starvation, while increased after refeeding. The findings were consistent with some research ones suggesting that variations in nutritional conditions might affect fish health [19]. In this study, the VSI value of fish was significantly higher during starvation and lower after refeeding. Similar results appeared in Nile tilapia (45.08 ± 0.92 g) (starved for three weeks and refed for three weeks) [18], showing that the change of visceral mass was closely related to nutritional status. The relative size of the hepatopancreas in fish was connected with nutritional status, and the lower HSI value might have been due to malnutrition [15]. In this study, the fish exhibited a significantly lower HSI value after starvation, which was in agreement with previous research showing the significance of hepatopancreas fat reserves during fasting [32]. Furthermore, the restriction of protein synthesis caused by starvation might not be conducive to preventing the decomposition of hepatopancreas fat in fish [36]. Nevertheless, the HSI value of *O. macrolepis* was significantly improved after refeeding. These findings demonstrated that the restoration of normal feeding met the nutritional requirements of the fish, lifted the restriction of protein synthesis and resulted in the increase of the hepatopancreas’s size. In the present study, the IPFI value of fish was significantly reduced during fasting. These findings could be explained by the rapid decomposing abilities of fish to break down the hepatopancreas fat and intraperitoneal fat into energy during the initial phases of fasting [37]. However, the nutrients in the visceral mass were exhausted with the starvation time extended, and the other energy storage parts of fish had to be mobilized to sustain life. 

Starvation and refeeding are recognized as having powerful impacts on the physiology features of fish. Serum biochemical factors are pressure-sensitive indexes, which can be used to indicate the physiological characteristics and health status of fish under pressure conditions [12]. TG and T-CHOL concentrations are important evaluation indicators that can be employed to reflect the nutritional states of fish [16]. Triglycerides are regarded as the most readily available form of fat storage during starvation-induced steatolysis [38]. As an important fat source, triglycerides migrate to vital tissues of the body during the early phase of fasting to maintain metabolic homeostasis. In this study, there was a significant decrease observed in serum TG and T-CHOL concentrations during starvation. Similar results were found in brown trout (29.7 ± 0.9 g), suggesting that the decline of plasma TG and T-CHOL concentrations was induced by starvation pressure (starvation for 7, 14, 28, 35 or 42 days) [16]. In addition, four weeks of starvation could lead to a decline in plasma cholesterol and TG of great sturgeon [15]. On the other hand, fish could suppress adipogenesis by inhibiting the activity of lipogenic enzymes, thereby reducing serum TG levels [14]. In the present study, the serum HDL and LDL concentrations of fish were reduced during fasting, which was inconsistent with what was previously reported, suggesting that starvation had an inhibitory effect on blood lipoprotein indicators of fish [16]. Due to a nutrient deficiency, starvation could suppress adipogenesis and promote steatolysis in fish [14]. This might be the reason for the decrease in serum lipids and lipoprotein levels of *O. macrolepis* during starvation. This study showed that significantly higher levels of TG and T-CHOL were discovered in serum after three weeks of refeeding. The results were consistent with those of Arslan et al. [16], who reported that restoring food supply was beneficial to the improvement of blood lipids levels in fish.

Protease is mainly responsible for hydrolyzing protein, and lipase activity reflects the utilization of lipids in the body [17]. During starvation, the amylase, lipase and protease activities showed a significant decline trend in *O. macrolepis*. Similar findings have been reported on *Megalobrama pellegrini* (fasting for 2, 4, 7 or 14 days) [34] and Songpu mirror carp (190.35 ± 1.36 g) (fasting for three or four weeks) [20]. The reduction of the digestive enzyme activity is helpful to reduce the metabolism capacity of fish. This is a protective mechanism against an excessive loss of energy to help fish deal with starvation stress [19]. After the resumption of feeding, the digestive enzymes activities of *O. macrolepis* improved strongly. This finding was similar to that of Gisbert et al. [39], who reported that prolonged food deprivation did not permanently impair digestive function of European glass eels (*Anguilla anguilla*), their digestive capacity rapidly recovered upon refeeding.

As is known, the immunity of fish is connected with food intake [22]. LYZ participates in nonspecific immune processes and is bound up with the immune function of the body [32]. In the present study, the levels of LYZ, ACP and ALP in the hepatopancreas of *O. macrolepis* presented an escalating trend in the starvation stage. This was in keeping with the work of Liu et al. [19], which discovered that the higher immune enzymes activities might be beneficial to prevent immune damage induced by starvation in fish. ACP plays an important role in immunologic processes including defense function and immunity moderation [40]. In fish, ALP has been regarded as a clinical marker for hepatic damage or disorders. The results showed that there was a significantly higher activity of ALP in the intestines of *O. macrolepis* after starvation. Conversely, a previous study showed that malnourished (two weeks of food deprivation) roach (*Rutilus rutilus caspicus*) (1.68 ± 0.12 g) tended to have a lower intestinal ALP activity [41]. 

ROS are composed of free radicals (oxygen-derived) and their associated non-free-radical active species such as H_2_O_2_. Oxidative stress occurs when the balance between ROS production and antioxidant defense mechanism is disrupted [26]. The immune system and antioxidant enzymes play an important role in the regulation of oxidation status. In the current study, the SOD, GSH-PX and CAT activities both in the hepatopancreas and intestines showed an increasing trend during starvation. The finding was in agreement with previous ones indicating that food scarcity could activate the antioxidant defense mechanisms of fish [23]. The degree of lipid peroxidation can be measured by the MDA concentration. The MDA concentration of *O. macrolepis* diminished after starvation, indicating that the lipid peroxidation level was lower. These results suggest that the enhanced antioxidant capacity may not only be due to the increment of antioxidant enzyme activity, but also to the reduction of lipid peroxidation derived from oxidative stress [18]. Moreover, this indicated that the improvement of the GSH-PX level had a sufficient detoxification effect on MDA after starvation. These results suggest that short-term starvation might protect against oxidative stress by regulating endogenous antioxidants in fish. After three weeks of refeeding, there was no significant difference in the hepatic GSH-PX activity of *O. macrolepis* between the starved–refed and the control groups, indicating that the peroxides decreased during the refeeding process. In the present study, the MDA concentration in the hepatopancreas appeared to have an upward trend after refeeding, suggesting that food reintroduction might induce lipid peroxidation [19]. Nevertheless, lipid peroxidation was effectively prevented by high levels of SOD and CAT in the liver of the starved–refed group after three weeks of re-feeding, so the MDA content was still lower than that in the control group [25].

Compared with proteins, fat stores were considered to be more readily mobilized to generate energy during the early stages of starvation [9]. Triglycerides are thought to be an accessible form of fat storage after steatolysis, and HSL participates in part of their hydrolysis reaction process [42]. This study showed that the TG content in tissues of *O. macrolepis* decreased significantly after starvation. In the present study, the HSL transcription levels were significantly upregulated in tissues after starvation, which was consistent with what was reported in Gibel carp (111.13 ± 0.65 g), indicating that the lipolysis potential was significantly enhanced after 21 days of food deprivation [14]. Furthermore, fatty acids are generated from lipid reserves and are mobilized for energy during food deprivation. CPT-1A is an important gene involved in the regulation of fatty acid oxidation. In this study, the expression levels of CPT-1A were significantly upregulated in tissues after starvation. This result was consistent with previous studies, suggesting that as an extra energy source, the fatty acids’ oxidative potential was activated, particularly the β-oxidation of mitochondria and peroxisomes [43]. These findings suggested that fat reserves were decomposed to provide energy fuel for *O. macrolepis* during the starvation phase. In fish, SREBP1 is a major regulator of the lipid biosynthetic pathway and FAS is a vital lipogenic enzyme in fatty acid de novo production process [44,45]. In this study, the expression levels of SREBP1 and FAS in tissues declined during starvation. The results demonstrated that the inhibition of the lipogenic enzyme activity directly associated with the reduction of triglyceride levels in fish. Despite its intrinsic limitations, the detection values of TG and lipoprotein are frequently utilized to deduce fat mobilization in response to starvation [1]. As mentioned above, serum lipid levels were significantly reduced during starvation. This result is in accordance with some research ones indicating that the deprivation of food and the suppression of the fatty acid synthesis commonly lead to a decline in serum triglycerides under starvation conditions [14]. 

In the present study, the TG content gradually recovered after refeeding. Meanwhile, the relative expression levels of SREBP1 and FAS in the hepatopancreas were similar to those in the control group. These results indicated that the recovery of the TG content might be mediated by the upregulation of the expression levels of lipogenesis genes. However, there are limitations to our results, which relied solely on three weeks of starvation. The relationship between intestinal microflora of this species and hunger needs further research.

## 5. Conclusions

In conclusion, the effects of fasting and refeeding on growth, digestion, nonspecific immunity and lipid metabolism were investigated in *O. macrolepis*. The results indicated that starvation inhibited the growth of *O. macrolepis*, and refeeding could improve the growth performance. It was found that *O. macrolepis* fish could respond to changes in nutritional status by changing their antioxidant capacity during starvation and refeeding. Lipid consumption conduced to sustain the fundamental energy requirements of *O. macrolepis* when food was scarce. Lipid energy mobilization was maintained by promoting adipolysis and fatty acid oxidation processes, as well as suppressing fat synthesis. The reintroduction of the food supply accelerated the recovery of fat levels. Understanding the duration of physiological and metabolic changes in *O. macrolepis* and their reversibility or irreversibility to supplementary feeding response could provide a valuable reference for the adaptability of *O. macrolepis* in large-scale culturing, proliferation and release.

## Figures and Tables

**Table 1:** Formulation of the commercial feed for *O. macrolepis* exposed to starvation and refeeding periods.

Component	Quantity
Crude protein	≥35%
Crude fat	≥6%
Crude fiber	≤9%
Crude ash	≤15%
Calcium	0.5–3.5%
Total phosphorus	0.8–2.0%
Lysine	≥1.6%
Moisture	≤10%

Multivitamins and minerals were also added to commercial diets, such as vitamins (A, B1, B2, C, D3 and E), magnesium sulfate and copper sulfate (Shandong Dehai Biotechnology Co., Ltd., Zaozhuang, China). However, manufacturers did not mark the quantity of these minerals.

**Table 2:** Primers used for fluorescence quantification for *O. macrolepis* exposed to starvation and refeeding periods.

Genes	Sequence (5′–3′)	Accession No.	Amplicon Size (bp)	Amplification Efficiency (%)
HSL ^A^	F:GCTTCACCATCCAGACTGCTCAC	MG735215.1	109	98.4
	R:CTGGCTGCACTGACTGACAACTC			
CPT-1A ^B^	F:CTCAGACGGTGTTCAGTGCCATC	MH553647	105	97.1
	R:TCCAGCCGTGATAGGACAAGAGG			
SREBP1 ^C^	F:GGCGACAAGAACCTCACTGATGG	MG735210.1	177	95.0
	R:GACAACCGACGACCACCACTTC			
FAS ^D^	F:ATCCACAGAGCCACCATCCTACC	MG735211.1	145	97.8
	R:CAAGTCCAGCATCCTCCAAGACAC			
β-Actin	F:TGACCCACACTGTACCCATC	JN254630.1	154	97.3
	R:CGGACAATTTCACTCTCGGC			

^A^ HSL, hormone-sensitive lipase. ^B^ CPT-1A, carnitine palmitoyl transferase 1 isoform A. ^C^ SREBP1, sterol regulatory element binding protein 1. ^D^ FAS, fatty acid synthase.

**Table 3:** Growth and biological indicators of *O. macrolepis* exposed to starvation and refeeding periods.

Starvation Period							Refeeding Period						
				(*p* Value)							(*p* Value)		
	Control	Starved/Refed	Control vs. Starved/Refed	Feeding Condition	Time	Interaction		Control	Starved/Refed	Control vs. Starved/Refed	Feeding Condition	Time	Interaction
IW (g)	2.50 ± 0.14	2.51 ± 0.17	ns	ns	ns	ns							
BW (g)				<0.001	<0.001	<0.001	BW (g)				<0.001	<0.001	ns
Weeks							Weeks						
1	3.61 ± 0.14 ^c^	2.10 ± 0.08 ^a^	**				4	6.67 ± 0.22 ^c^	2.86±0.10 ^c^	**			
2	4.57 ± 0.35 ^b^	1.72 ± 0.09 ^ab^	**				5	7.80 ± 0.15 ^b^	4.40 ± 0.21 ^b^	**			
3	5.72 ± 0.09 ^a^	1.44 ± 0.07 ^b^	**				6	8.95 ± 0.39 ^a^	5.49 ± 0.13 ^a^	**			
CF (%)				<0.001	ns	<0.01	CF (%)				<0.001	<0.001	ns
Weeks							Weeks						
1	1.67 ± 0.07	1.47 ± 0.08 ^a^	*				4	1.89 ± 0.07 ^c^	1.37 ± 0.05 ^c^	**			
2	1.72 ± 0.10	1.3 ± 0.05 ^ab^	**				5	1.98 ± 0.04 ^b^	1.55 ± 0.04 ^b^	**			
3	1.85 ± 0.15	1.17 ± 0.02 ^b^	**				6	2.09 ± 0.06 ^a^	1.63 ± 0.08 ^a^	**			
VSI ^d^ (%)				<0.001	ns	<0.001	VSI ^d^ (%)				<0.001	<0.01	ns
Weeks							Weeks						
1	5.64 ± 0.11 ^c^	4.91 ± 0.06 ^a^	**				4	6.94 ± 0.39 ^c^	4.65 ± 0.41 ^c^	**			
2	5.98 ± 0.05 ^b^	4.26 ± 0.06 ^b^	**				5	7.69 ± 0.52 ^ab^	5.15 ± 0.13 ^ab^	**			
3	6.35 ± 0.10 ^a^	3.96 ± 0.18 ^c^	**				6	8.02 ± 0.46 ^a^	5.52 ± 0.17 ^a^	**			
HSI ^e^ (%)				<0.001	<0.001	<0.001	HSI ^e^ (%)				<0.001	<0.001	ns
Weeks							Weeks						
1	1.81 ± 0.10 ^b^	1.58 ± 0.05 ^a^	*				4	2.13 ± 0.03 ^c^	1.16 ± 0.03 ^c^	**			
2	1.95 ± 0.04 ^ab^	1.37 ± 0.07 ^b^	**				5	2.25 ± 0.04 ^ab^	1.28 ± 0.08 ^ab^	**			
3	2.04 ± 0.04 ^a^	0.93 ± 0.08 ^c^	**				6	2.33 ± 0.05 ^a^	1.34 ± 0.03 ^a^	**			
IPFI ^f^ (%)				<0.001	<0.001	<0.001	IPFI ^f^ (%)				<0.001	<0.001	ns
Weeks							Weeks						
1	1.75 ± 0.07^b^	1.43 ± 0.05 ^a^	**				4	2.05 ± 0.05 ^c^	0.81 ± 0.02 ^c^	**			
2	1.83 ± 0.09 ^ab^	1.16 ± 0.06 ^b^	**				5	2.12 ± 0.03 ^b^	0.90 ± 0.09 ^b^	**			
3	1.92 ± 0.05 ^a^	0.70 ± 0.03 ^c^	**				6	2.19 ± 0.04 ^a^	1.03 ± 0.04 ^a^	**			

Different superscript letters in the same column indicate a significant difference between successive times (*p* < 0.05). Significant differences between the two groups at each sampling time are identified with different markers (* *p* < 0.05; ** *p* < 0.01; ns, no significant difference). ^a^ IW, initial weight. ^b^ BW, body weight. ^c^ CF, condition factor (%) = body weight (g) × 100/ body length^3^ (cm). ^d^ VSI, viscera index (%) = viscera weigh × 100/ body weight. ^e^ HSI, hepatopancreas index (%) = hepatopancreas weigh × 100/ body weight. ^f^ IPFI, intraperitoneal fat index (%) = intraperitoneal fat weight × 100/ body weight.

**Table 4:** Serum parameters of *O. macrolepis* exposed to starvation and refeeding periods.

Starvation Period							Refeeding Period						
	Control	Starved/Refed	Control vs. Starved/Refed	(*p* Value)				Control	Starved/Refed	Control vs. Starved/Refed	(*p* Value)		
				Feeding Condition	Time	Interaction					Feeding Condition	Time	Interaction
TG (mmol/L)				<0.001	<0.001	<0.001	TG (mmol/L)				<0.001	<0.001	<0.001
Weeks							Weeks						
1	1.67 ± 0.05	1.46±0.07 ^a^	*				4	1.68 ± 0.02	1.19 ± 0.04 ^c^	**			
2	1.65 ± 0.12	1.10 ± 0.11 ^b^	**				5	1.69 ± 0.09	1.41 ± 0.05 ^b^	**			
3	1.69 ± 0.05	0.83 ± 0.05 ^c^	**				6	1.73 ± 0.07	1.67 ± 0.06 ^a^	ns			
T-CHOL (mmol/L)				<0.001	<0.01	<0.01	T-CHOL (mmol/L)				<0.001	<0.001	<0.001
Weeks							Weeks						
1	1.85 ± 0.08	1.63 ± 0.05 ^a^	*				4	1.87 ± 0.05	1.49 ± 0.06 ^c^	**			
2	1.87 ± 0.04	1.39 ± 0.07 ^b^	**				5	1.88 ± 0.07	1.74 ± 0.04 ^ab^	*			
3	1.86 ± 0.06	1.15 ± 0.14 ^c^	**				6	1.89 ± 0.06	1.86 ± 0.05 ^a^	ns			
HDL (mmol/L)				<0.001	ns	ns	HDL (mmol/L)				<0.01	ns	ns
Weeks							Weeks						
1	1.68 ± 0.10	1.35 ± 0.05	**				4	1.64 ± 0.10	1.41 ± 0.07	*			
2	1.65 ± 0.14	1.28 ± 0.11	**				5	1.66 ± 0.10	1.54 ± 0.05	*			
3	1.62 ± 0.06	1.13 ± 0.12	**				6	1.67 ± 0.08	1.57 ± 0.08	*			
LDL ^d^ (mmol/L)				<0.001	<0.05	ns	LDL ^d^ (mmol/L)				<0.01	ns	ns
Weeks							Weeks						
1	0.67 ± 0.06	0.57 ± 0.03 ^a^	**				4	0.66 ± 0.09	0.49 ± 0.05	*			
2	0.65 ± 0.03	0.47 ± 0.06 ^ab^	**				5	0.62 ± 0.05	0.54 ± 0.04	*			
3	0.63 ± 0.07	0.46 ± 0.05 ^b^	**				6	0.61 ± 0.09	0.58 ± 0.03	*			

Different superscript letters in the same column indicate a significant difference between successive times (*p* < 0.05). Significant differences between the two groups at each sampling time are identified with different markers (* *p* < 0.05; ** *p* < 0.01; ns, no significant difference). ^a^ TG, triglyceride. ^b^ T-CHOL, total cholesterol. ^c^ HDL, high-density lipoprotein. ^d^ LDL, low-density lipoprotein.

**Table 5:** Intestinal digestive enzyme activities of *O. macrolepis* exposed to starvation and refeeding periods.

Starvation Period							Refeeding Period						
	Control	Starved/Refed	Control vs. Starved/ Refed	(*p* Value)				Control	Starved/ Refed	Control vs. Starved/Refed	(*p* Value)		
				Feeding Condition	Time	Interaction					Feeding Condition	Time	Interaction
Amylase (U/mgprot)				<0.001	<0.001	ns	Amylase (U/mgprot)				<0.01	ns	<0.05
Weeks							Weeks						
1	0.34 ± 0.05	0.21 ± 0.02 ^a^	**				4	0.32 ± 0.09	0.15 ± 0.01 ^b^	*			
2	0.38 ± 0.06	0.17 ± 0.02 ^ab^	**				5	0.22 ± 0.06	0.18 ± 0.01 ^b^	ns			
3	0.24 ± 0.06	0.09 ± 0.01 ^c^	**				6	0.28 ± 0.03	0.26 ± 0.03 ^a^	ns			
Lipase (U/mgprot)				<0.01	<0.001	<0.001	Lipase (U/mgprot)				<0.001	<0.001	<0.01
Weeks							Weeks						
1	27.32 ± 1.71	33.70 ± 2.81 ^a^	*				4	31.41 ± 2.05	20.34 ± 2.04 ^c^	**			
2	28.98 ± 1.02	24.85 ± 1.15 ^b^	*				5	32.50 ± 1.95	25.90 ± 1.33 ^b^	*			
3	29.64 ± 2.32	17.87 ± 1.82 ^c^	**				6	33.31 ± 1.72	31.94 ± 0.92 ^a^	ns			
Protease (U/mgprot)				<0.001	ns	<0.05	Protease (U/mgprot)				<0.05	ns	ns
Weeks							Weeks						
1	11.01 ± 1.07 ^b^	10.23 ± 0.56	ns				4	13.05 ± 1.00	9.72 ± 1.53	*			
2	13.42 ± 1.49 ^a^	9.53 ± 0.47	*				5	12.48 ± 1.77	10.87 ± 1.03	*			
3	13.54 ± 1.30 ^a^	8.71 ± 0.56	*				6	12.91 ± 1.64	13.36 ± 1.04	*			

Different superscript letters “a, b and c” in the same column indicate a significant difference between successive times (*p* < 0.05). Significant differences between the two groups at each sampling time are identified with different markers (* *p* < 0.05; ** *p* < 0.01; ns, no significant difference).

**Table 6:** Hepatic immune enzyme activities of *O. macrolepis* exposed to starvation and refeeding periods.

Starvation Period							Re-Feeding Period						
	Control	Starved/Refed	Control vs. Starved/Refed	(*p* Value)				Control	Starved/Refed	Control vs. Starved/Refed	(*p* Value)		
				Feeding Condition	Time	Interaction					Feeding Condition	Time	Interaction
LYZ (U/mL)				<0.001	ns	<0.01	LYZ (U/mL)				<0.05	ns	ns
Weeks							Weeks						
1	0.75 ± 0.04	0.88 ± 0.05 ^b^	*				4	0.71 ± 0.07	0.89 ± 0.03	*			
2	0.70 ± 0.05	1.09 ± 0.08 ^a^	**				5	0.75 ± 0.09	0.85 ± 0.14	*			
3	0.75 ± 0.04	0.96 ± 0.04 ^b^	**				6	0.75 ± 0.12	0.79 ± 0.07	*			
ACP (U/mgprot)				<0.001	<0.01	<0.001	ACP (U/mgprot)				ns	ns	ns
Weeks							Weeks						
1	0.85 ± 0.04	0.92 ± 0.03 ^b^	ns				4	0.86 ± 0.08	0.90 ± 0.04	ns			
2	0.82 ± 0.07	1.17 ± 0.06 ^a^	**				5	0.87 ± 0.04	0.91 ± 0.04	ns			
3	0.85 ± 0.03	0.96 ± 0.02 ^b^	*				6	0.84 ± 0.05	0.90 ± 0.04	ns			
ALP (U/mgprot)				<0.001	<0.01	ns	ALP (U/mgprot)				ns	ns	ns
Weeks							Weeks						
1	0.61 ± 0.04	0.72 ± 0.05 ^b^	**				4	0.66 ± 0.06	0.69 ± 0.05	ns			
2	0.66 ± 0.05	0.85 ± 0.05 ^a^	**				5	0.67 ± 0.04	0.65 ± 0.05	ns			
3	0.65 ± 0.05	0.75 ± 0.03 ^ab^	**				6	0.71 ± 0.05	0.66 ± 0.04	ns			

Different superscript letters in the same column indicate a significant difference between successive times (*p* < 0.05). Significant differences between the two groups at each sampling time are identified with different markers (* *p* < 0.05; ** *p* < 0.01; ns, no significant difference). ^a^ LYZ, lysozyme. ^b^ ACP, acid phosphate. ^c^ ALP, alkaline phosphate.

**Table 7:** Intestinal immune enzyme activities of *O. macrolepis* exposed to starvation and refeeding periods.

Starvation Period							Re-Feeding Period						
	Control	Starved/Refed	Control vs. Starved/Refed	(*p* Value)				Control	Starved/Refed	Control vs. Starved/Refed	(*p* Value)		
				Feeding Condition	Time	Interaction					Feeding Condition	Time	Interaction
LYZ (U/mL)				<0.001	<0.001	<0.01	LYZ (U/mL)				<0.01	ns	ns
Weeks							Weeks						
1	0.54 ± 0.01	0.66 ± 0.04 ^b^	**				4	0.55 ± 0.04	0.61 ± 0.01	*			
2	0.52 ± 0.03	0.63 ± 0.02 ^b^	**				5	0.53 ± 0.09	0.64 ± 0.04	*			
3	0.56 ± 0.03	0.82 ± 0.03 ^a^	**				6	0.52 ± 0.09	0.63 ± 0.02	*			
ACP (U/mgprot)				<0.01	ns	ns	ACP (U/mgprot)				<0.01	ns	ns
Weeks							Weeks						
1	0.67 ± 0.12	0.77 ± 0.03	*				4	0.61 ± 0.06	0.71 ± 0.04	*			
2	0.64 ± 0.08	0.73 ± 0.03	*				5	0.62 ± 0.10	0.72 ± 0.05	*			
3	0.66 ± 0.04	0.83 ± 0.03	*				6	0.64 ± 0.07	0.74 ± 0.07	*			
ALP (U/mgprot)				<0.001	<0.05	ns	ALP (U/mgprot)				ns	ns	ns
Weeks							Weeks						
1	0.45 ± 0.09	0.56 ± 0.02 ^ab^	**				4	0.52 ± 0.02	0.59 ± 0.11	ns			
2	0.43 ± 0.02	0.52 ± 0.02 ^b^	**				5	0.52 ± 0.01	0.53 ± 0.04	ns			
3	0.45 ± 0.02	0.63 ± 0.02 ^a^	**				6	0.52 ± 0.01	0.50 ± 0.03	ns			

Different superscript letters in the same column indicate a significant difference between successive times (*p* < 0.05). Significant differences between the two groups at each sampling time are identified with different markers (* *p* < 0.05; ** *p* < 0.01; ns, no significant difference). ^a^ LYZ, lysozyme. ^b^ ACP, acid phosphate. ^c^ ALP, alkaline phosphate.

**Table 8:** Hepatic antioxidant indices of *O. macrolepis* exposed to starvation and refeeding periods.

Starvation Period							Re-Feeding Period						
	Control	Starved/Refed	Control vs. Starved/Refed	(*p* Value)				Control	Starved/Refed	Control vs. Starved/Refed	(*p* Value)		
				Feeding Condition	Time	Interaction					Feeding Condition	Time	Interaction
SOD (U/mgprot)				<0.001	<0.05	<0.01	SOD (U/mgprot)				<0.001	ns	ns
Weeks							Weeks						
1	84.15 ± 2.14	97.42 ± 2.03 ^b^	*				4	79.25 ± 5.89	100.64 ± 4.86	**			
2	81.04 ± 5.24	112.09 ± 2.90 ^a^	**				5	78.64 ± 3.47	94.73 ± 1.61	**			
3	76.46 ± 4.55	104.11 ± 3.68 ^ab^	**				6	74.4 ± 4.37	90.59 ± 6.64	**			
CAT (U/mgprot)				<0.001	<0.01	<0.01	CAT (U/mgprot)				<0.001	<0.001	ns
Weeks							Weeks						
1	39.43 ± 1.88	47.32 ± 2.60 ^b^	*				4	35.55 ± 2.06	45.47 ± 2.41 ^a^	**			
2	37.82 ± 1.08	59.53 ± 1.68 ^a^	**				5	33.22 ± 2.06	40.13 ± 1.97 ^b^	**			
3	36.37 ± 2.56	54.28 ± 3.76 ^a^	**				6	31.97 ± 1.07	36.27 ± 1.12 ^b^	**			
GSH-PX (U/mgprot)				<0.001	<0.001	<0.01	GSH-PX (U/mgprot)				<0.001	ns	<0.05
Weeks							Weeks						
1	63.13 ± 1.01	78.36 ± 1.92 ^c^	**				4	71.84 ± 1.82	83.99 ± 2.64 ^a^	**			
2	64.53 ± 2.84	88.47 ± 1.86 ^a^	**				5	72.62 ± 1.36	80.52 ± 1.91 ^ab^	*			
3	66.63 ± 1.44	82.87 ± 1.16 ^b^	**				6	73.50 ± 2.12	77.77 ± 3.44 ^b^	*			
MDA ^d^ (U/mgprot)				<0.001	<0.001	<0.001	MDA ^d^ (U/mgprot)				<0.001	<0.05	<0.01
Weeks							Weeks						
1	4.74 ± 0.17	2.89 ± 0.28 ^a^	**				4	4.51 ± 0.29	1.37 ± 0.09 ^b^	**			
2	4.67 ± 0.13	1.74 ± 0.21 ^b^	**				5	4.65 ± 0.17	1.53 ± 0.19 ^b^	**			
3	4.45 ± 0.21	0.70 ± 0.18 ^c^	**				6	4.35 ± 0.21	2.53 ± 0.39 ^a^	**			

Different superscript letters in the same column indicate a significant difference between successive times (*p* < 0.05). Significant differences between the two groups at each sampling time are identified with different markers (* *p* < 0.05; ** *p* < 0.01; ns, no significant difference). ^a^ SOD, superoxide dismutase. ^b^ CAT, catalase. ^c^ GSH-PX, glutathione peroxidase. ^d^ MDA, malondialdehyde.

**Table 9:** Intestinal antioxidant indices of *O. macrolepis* exposed to starvation and refeeding periods.

Starvation Period							Refeeding Period						
				(*p* Value)							(*p* Value)		
	Control	Starved/Refed	Control vs. Starved/Refed	Feeding Condition	Time	Interaction		Control	Starved/Refed	Control vs. Starved/Refed	Feeding Condition	Time	Interaction
SOD (U/mgprot)				<0.001	<0.05	<0.01	SOD (U/mgprot)				<0.001	ns	ns
Weeks							Weeks						
1	40.34 ± 2.04	53.71 ± 1.80 ^b^	**				4	40.74 ± 1.4	55.13 ± 1.35	**			
2	41.39 ± 2.15	53.31 ± 2.96 ^b^	**				5	41.60 ± 2.21	51.21 ± 1.90	**			
3	40.51 ± 2.13	61.18 ± 1.59 ^a^	**				6	41.09 ± 1.11	50.45 ± 2.28	**			
CAT (U/mgprot)				<0.001	<0.01	<0.01	CAT (U/mgprot)				<0.001	ns	ns
Weeks							Weeks						
1	10.81 ± 1.05	15.92 ± 1.06 ^b^	**				4	10.48 ± 1.63	20.79 ± 1.78	**			
2	10.75 ± 1.40	18.77 ± 1.12 ^b^	**				5	10.67 ± 1.98	16.90 ± 1.33	**			
3	10.07 ± 1.59	22.65 ± 1.42 ^a^	**				6	10.62 ± 1.22	16.23 ± 1.72	**			
GSH-PX (U/mgprot)				<0.001	<0.01	<0.001	GSH-PX (U/mgprot)				<0.001	ns	ns
Weeks							Weeks						
1	26.60 ± 1.57	30.22 ± 1.61 ^b^	*				4	26.44 ± 1.63	36.08 ± 1.23	**			
2	26.30 ± 1.01	40.71 ± 2.42 ^a^	**				5	27.13 ± 2.97	32.16 ± 3.44	**			
3	26.73 ± 1.29	40.23 ± 2.60 ^a^	**				6	26.30 ± 2.58	28.66 ± 2.34	**			
MDA ^d^ (U/mgprot)				<0.001	<0.01	<0.05	MDA ^d^ (U/mgprot)				<0.001	ns	ns
Weeks							Weeks						
1	6.70 ± 0.35	4.4 ± 0.48 ^a^	*				4	6.33 ± 1.11	3.17 ± 0.25	**			
2	6.43 ± 1.08	2.56 ± 0.56 ^b^	**				5	6.45 ± 0.56	3.14 ± 0.64	**			
3	6.39 ± 0.69	1.58 ± 0.31 ^bc^	**				6	6.67 ± 1.32	2.97 ± 0.84	**			

Different superscript letters in the same column indicate a significant difference between successive times (*p* < 0.05). Significant differences between the two groups at each sampling time are identified with different markers (* *p* < 0.05; ** *p* <0.01; ns, no significant difference). ^a^ SOD, superoxide dismutase. ^b^ CAT, catalase. ^c^ GSH-PX, glutathione peroxidase. ^d^ MDA, malondialdehyde.

## Data Availability

All data are included in the article.
